# Red Upconverter Nanocrystals Functionalized with Verteporfin for Photodynamic Therapy Triggered by Upconversion

**DOI:** 10.3390/ijms23136951

**Published:** 2022-06-22

**Authors:** Ivana Miletto, Chiara Gionco, Maria Cristina Paganini, Erik Cerrato, Leonardo Marchese, Enrica Gianotti

**Affiliations:** 1 Dipartimento di Scienze del Farmaco, Università del Piemonte Orientale, Largo Donegani 2/3, 28100 Novara, Italy; 2 Dipartimento di Chimica, Università degli Studi di Torino, Via Pietro Giuria 9, 10125 Torino, Italy; c.gionco@inrim.it (C.G.); mariacristina.paganini@unito.it (M.C.P.); e.cerrato@inrim.it (E.C.); 3 Dipartimento di Scienze ed Innovazione Tecnologica, Università del Piemonte Orientale, Viale Teresa Michel 11, 15121 Alessandria, Italy; leonardo.marchese@uniupo.it; 4 Dipartimento per la Sostenibilità e la Transizione Ecologica, Università del Piemonte Orientale, Piazza Sant’Eusebio 5, 13100 Vercelli, Italy

**Keywords:** red upconverter nanoparticles, upconversion, photodynamic therapy, theranostic, optical imaging, core–shell nanoparticles, lanthanide doped nanoparticles

## Abstract

Upconversion (UC) nanoparticles characterized by red upconversion emission, particularly interesting for biological applications, have been prepared and subsequently modified by the covalent anchoring of Verteporfin (Ver), an FDA approved photosensitizer (PS) which usually exerts its photodynamic activity upon excitation with red light. ZrO_2_ was chosen as the platform where Yb^3+^ and Er^3+^ were inserted as the sensitizer and activator ions, respectively. Careful control of the doping ratio, along with a detailed physico-chemical characterization, was carried out. Upon functionalization with a silica shell to covalently anchor the photosensitizer, a theranostic nanoparticle was obtained whose architecture, thanks to a favorable energy level match and a uniform distribution of the PS, allowed us to trigger the photodynamic activity of Ver by upconversion, thus paving the way to the use of Photodynamic Therapy (PDT) in deep tissues, thanks to the higher penetrating power of NIR light.

## 1. Introduction

The design of appropriately functionalized nanomaterials allows the development of highly selective platforms capable of integrating imaging and therapy. Theranostic, e.g., the coupling of therapy and diagnostic, is gaining more and more interest in the scientific community as it offers the great opportunity to integrate multiple techniques to arrive at a complete and personalized imaging/therapy regimen [1]. The development of theranostic agents mostly rely on the incorporation of therapeutic functions into molecular imaging contrast agents [2]; in this respect, the therapeutic role can be implemented in different ways. An effective coupling of imaging and therapy is represented by imaging-guided surgery, for tumor resection and post-surgery evaluation, where the precise visualization of malignant tissues is fundamental for ensuring the complete resection of the tumor [3,4]. More traditionally, therapeutic active moieties can be delivered and released to the target site by using nanometric carriers which are active in optical imaging [5,6,7,8] or magnetic resonance imaging [9,10,11]. As an alternative to drug delivery, the therapeutic agent can be generated in situ through photo activation, such as in Photodynamic Therapy (PDT), where a photosensitizer (PS) is able to generate cytotoxic reactive oxygen species once activated by illumination with light of proper wavelength [12,13,14,15,16], or in the case of Photothermal Therapy, where the nonradiative conversion of absorbed photon energy into heat causes cell death [17,18]. Photoluminescence (PL) is one of the most exploited detection techniques for a wide range of applications in biochemistry and molecular biology, including bioassays, medical diagnostics, and genomics [19]. Although representing a powerful noninvasive tool applicable to a plethora of biological samples, ranging from in vitro cultured cells and ex vivo tissues to in vivo imaging of living organisms, several limitations must be considered. Most of the conventional and most studied fluorescent contrast agents are represented by organic fluorophores [20,21], fluorescent proteins [22], organometallic complexes [23], organic or inorganic nanoparticles [24,25], and semiconductor quantum dots [26]; in these classes of PL agents, fluorescence emission is generated by a conventional down-conversion mechanism by using ultraviolet (UV) or visible excitation wavelength. Therefore, fluorescence signals often are found in the Visible region, where autofluorescence and light scattering of biological samples is high, thus resulting in a poor signal-to-background ratio [27]. Moreover, the low penetration depth of UV and Vis light limit the application of PL based detection to in vitro applications or superficial tissues. The use of NIR (near-infrared) excitation light would allow for better exploitation of the “optical transparency windows” of biological tissues [28], the so-called “first biological window” (650–950 nm) and the more interesting “second biological window” (1000–1500 nm), where light offers deeper penetration and reduced optical scattering, along with low autofluorescence of biological samples. In this scenario, a lanthanide-based compound and nanomaterials are attracting more and more interest due to their outstanding optical properties, e.g., emission in the Vis and NIR region, narrow absorption and emission bands, long excited state lifetimes and high emission efficiency, arising from the electron configuration of Ln^3+^ ions, characterized by an incompletely filled 4f shell, shielded from the local microenvironment by the external complete 5s^2^ and 5p^6^ shells [29,30]. Moreover, upconversion (UC) emission is particularly interesting for NIR bioimaging, as through this phenomenon two or more lower-energy photons (e.g., NIR photons) are converted into a single emitted high-energy photon (e.g., Vis photon). When lanthanide ions are doped into a suitable host material, the UC process is favored. To achieve high upconversion efficiency in upconverter nanoparticles (UCNP), sensitizer ions must be co-doped with activator ions having closely matched excited states. The most studied sensitizers are Yb^3+^ and Nd^3+^, which collect and transfer energy to activators, e.g., Er^3+^, Tm^3+^, and Ho^3+^, which emit green and red, red, and blue light, respectively. One of the most used UC pairs is represented by Yb^3+^–Er^3+^, where the green and red emissions of Er^3+^ are obtained through UC mechanism. However, preferential red emission, with respect to green, is desired for biological applications; therefore, most efforts have been focused on the development of UCNPs characterized by a strong and single-band red emission from the Yb–Er couple under the NIR irradiation [31]. Besides the dopants, the correct choice of the host matrix is fundamental, as it provides a platform for the energy transfer. A huge number of host materials has been tested, e.g., fluorides [32], vanadates [33], oxysulfide, oxychloride, oxyfluorides [34,35], and different oxides such as Y_2_O_3_ [36] and ZrO_2_ [29,37,38]. In particular, beside biocompatible oxide hosts, zirconium dioxide (ZrO_2_) is considered a particularly interesting material for optical application, as it is characterized by good transparency, a wide bandgap, high refractive index, and low phonon energy (470–500 cm^−1^), which render it an ideal host for Ln^3+^ ions in the preparation of UCNPs.

Herein, we report the preparation and characterization of theranostic nanoparticles for PDT and imaging, where the singlet oxygen (^1^O_2_) delivery is activated through a UC mechanism. Current photosensitizers for PDTs have, as their main limitation, the need of Visible light to be activated, thus limiting their applications due to the poor penetrating power of Visible light. The possibility of exploiting the phenomenon of UC in UCNPs capable of converting near-infra-red excitation into visible light, for the subsequent excitation of a PS, is of great interest [39], thus expanding the potential application of PDT to deeper tissues.

For this purpose, red upconverter nanoparticles were prepared by co-doping of ZrO_2_ nanoparticles with Yb^3+^ and Er^3+^ as the sensitizer and activator ions, respectively. Three different Yb^3+^ loadings were investigated, and a detailed physico-chemical characterization was carried out to identify the best performing sample to be coated with an amino-modified silica shell for the covalent anchoring of verteporfin as the photosensitizer for PDT.

In Table 1, acronyms, composition, and description of the pristine and modified samples are reported.

## 2. Results

### Synthesis and Characterization of Pristine ZrO_2_ Nanocrystals Co-Doped with Er^3+^ and Yb^3+^ Ions

**Figure 1:**
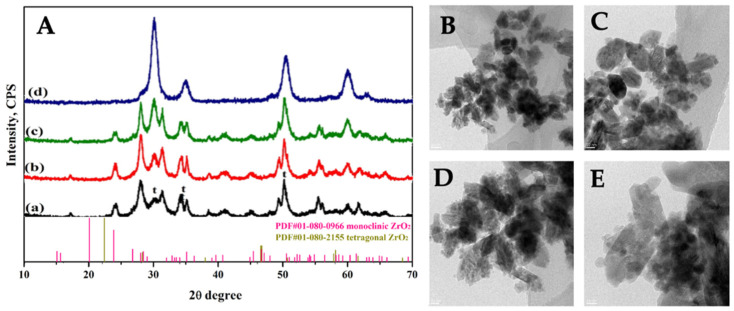
(**A**). XRD patterns of pure ZrO_2_ (curve a), ZEY2 (curve b), ZEY5 (curve c), and ZEY10 (curve d) (**B**–**E**). Representative TEM images of pure ZrO_2_, ZEY2, ZEY5, and ZEY10.

A facile hydrothermal route [40,41] was applied for the preparation of ZrO_2_ nanocrystals co-doped with Er^3+^ and Yb^3+^. Three samples were prepared with a fixed Er^3+^ loading (0.5%) and a variable sensitizer Yb^3+^ loading (2%, 5%, and 10%). The actual loading of the lanthanide dopants was confirmed by EDX (energy Dispersive X-ray) analysis (see Table S1 in the Supplementary Materials). XRD patterns of the co-doped samples are reported in Figure 1A and contrasted to the pattern of pure ZrO_2_ sample prepared by the same method. As already reported [40,41], the analysis of the diffraction pattern of pure ZrO_2_ revealed the presence of a mixture of monoclinic and tetragonal phases, labelled as m-ZrO_2_ and t-ZrO_2_, respectively, in Figure 1. When increasing the lanthanide ion content zirconia phases change from monoclinic, which is the predominant phase in the low dopant content samples, to tetragonal, which became favored along with the increase in the loading. ZEY10, which is the highest loading sample investigated, is mainly constituted by t-ZrO_2_. The size and morphology of the zirconia crystals were investigated by Transmission Electron Microscopy (Figure 1B–E), showing that both pure ZrO_2_ and co-doped samples are mainly constituted of anisotropic monoclinic phase crystallites (ca. 50 × 20 nm) and smaller particles, typical of the tetragonal phase, are present.

**Figure 2:**
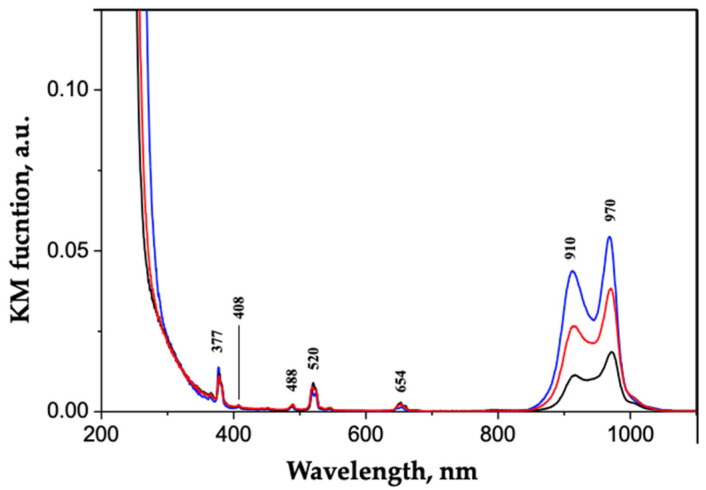
DR UV-vis-NIR spectra of ZEY2 (curve a, black), ZEY5 (curve b, red), and ZEY10 (curve c, blue).

The optical properties of the lanthanide doped zirconia samples were investigated by Diffuse Reflectance (DR) UV-Vis-NIR spectroscopy and steady-state fluorescence spectroscopy. In Figure 2, the DR UV-Vis-NIR spectra of the three co-doped samples are reported.

**Figure 3:**
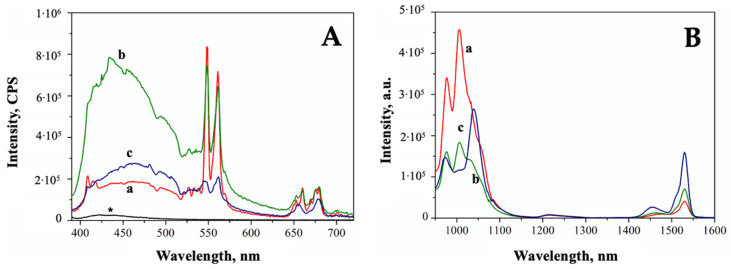
Fluorescence spectra of ZEY2 (curve a, red), ZEY5 (curve b, green), and ZEY10 (curve c, blue) in the Visible region (**A**) upon excitation at 377 nm and in the NIR region (**B**) upon excitation at 910 nm. Fluorescence spectrum of pure ZrO_2_ is reported in section A (black curve, *) for the sake of comparison.

The typical ZrO_2_ band-gap transition at 250 nm appears to be slightly affected by the presence of dopants only in the case of the ZEY10 sample, where the highest dopant loading was applied. In the Vis region, the absorption bands corresponding to Er^3+^ f-f transitions are present with the same intensity, as expected, as Er^3+^ loading was the same in all samples. On the contrary, the intensity of the Yb^3+^ absorption bands in the NIR range (800–1100 nm) increases along with the increase in the Yb^3^ loading. The Vis and NIR emission spectra of all samples are reported in Figure 3A,B, respectively; 377 nm and 910 nm were chosen as excitation wavelengths for Er^3+^ and Yb^3+^, respectively, on the basis of an excitation wavelength screening, as reported in Figure S1 in the Supplementary Materials. The typical emission of Er^3+^ in the green (548 nm, 561 nm) and in the red (660 nm, 679 nm) region of the spectrum is detected in all the samples, as well as the NIR component of Er^3+^ emissions at 1454 nm and 1531 nm. Although the samples were prepared with the same Er^3+^ loading and were characterized by almost completely overlapping DR UV-Vis-NIR spectra, the intensity of visible emissions due to Erbium decreases along with the increase in Yb^3+^ loading due to a quenching effect of Yb^3+^ ions on the emission of neighboring Er^3+^ ions.

A decrease in the emission of Yb^3+^ along with the increase in its concentration is observed as well, confirming that dopant concentrations exceeding 5% often result in quenched emissions, as already observed in the literature for similar systems [42]. Apart from the decrease in the emission intensity, a change in the relative intensities of the different component of the complex fluorescence band of Yb^3+^ is also detected. At least three components could be identified in the emission band of Yb^3+^ associated with the transitions from the lowest sublevel of excited multiplet ^2^F_5/2_ to the sublevels of ground multiplet ^2^F_7/2_ [43]. In particular, the most intense signals at 977 nm and 1005 nm are assigned to the transition from the sublevel 5 of the excited state ^2^F_5/2_ to the level 1 and 2 of the ground state ^2^F_7/2_, respectively. A change in the local environment of the lanthanide can be responsible of the differences in relative intensities.

**Figure 4:**
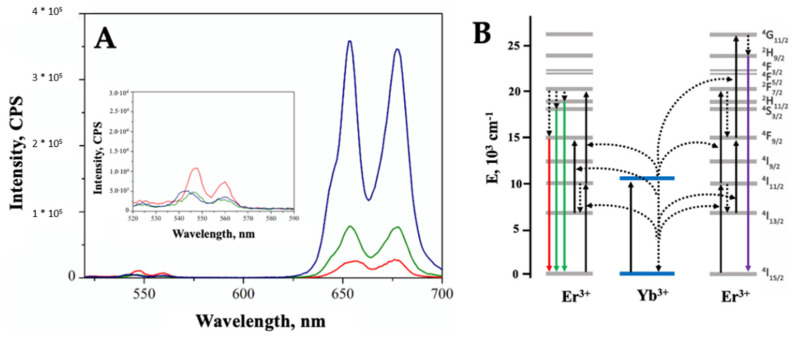
(**A**). UC emission spectra of ZEY2 (curve a, red), ZEY5 (curve b, green), and ZEY10 (curve c, blue) under NIR excitation (980 nm laser, power: 1.24 W); (**B**). Schematic illustration of UC processes and involved levels.

The effect of the sensitizer dopant (Yb^3+^), loading on the upconversion (UC) properties under NIR excitation of the co-doped samples was investigated by using a set-up equipped with a 980 nm diode-pumped-solid-state (DPSS) laser with variable power (0–2 W) as the source and a R928 photomultiplier as the detector. Two different power values were tested, 1.01 and 1.24 W, and the resulting UC emission spectra showed that the higher the laser power, the higher the intensity of UC (Figure S2 in the Supplementary Materials). The main components of the UC emission spectra obtained at 1.24 W (Figure 4A) are located in the red region of the spectrum (654 nm and 678 nm) whilst very low green UC is detected (548 nm and 560 nm, see inset of Figure 4).

The intensity of the red UC is much higher than green UC and increases along with the increase in Yb^3+^ loading, whilst an opposite trend was observed for the green UC. In order to fully understand this behavior, it is helpful to recall that the red emission of Er^3+^ originates from the transition ^4^F_9/2_ → ^4^I_15/2_ and the population mechanisms of the ^4^F_9/2_ level can be mainly: (i) direct population from the ^4^I_13/2_ level, which can be in turn populated through non radiative relaxation of ^4^I_11/2_ level; (ii) non-radiative relaxation from the ^4^S_3/2_ level; and (iii) cross relaxation process of ^4^F_7/2_ → ^4^F_9/2_ and ^4^I_11/2_ → ^4^F_9/2_ between two nearby Er^3+^ ions. Nevertheless, the absorption cross section of Yb^3+^ being much higher than that of Er^3+^ (under 980 nm excitation) and two levels quite even, the energy transfer (ET) from Yb^3+^ to Er^3+^ is highly efficient. Therefore, when increasing the Yb^3+^ concentration, higher populations of both ^4^F_7/2_ and ^4^I_11/2_ levels are induced by ET, thus increasing the probability of strong resonant cross relaxation between two nearby Er^3+^ ions (mechanism labelled as iii), resulting in a stronger red emission [44]. In addition, the enhancement in red emission can be also explained as a consequence of the decreased interatomic distances between Yb^3+^ and Er^3+^ ions (at high loadings) which favors energy back transfer (EBT) processes from Er^3+^ to Yb^3+^, leading to increased population of ^4^I_13/2_ [45,46].

Although the ESA mechanism (Excited State Absorption) is considered less efficient, it must be considered that Er^3+^-Er^3+^ UC can occur through this mechanism, as observed in the case of ZrO_2_ crystals doped with Er^3+^ at different concentrations (see Figure S3 in the Supplementary Materials). When Er^3+^ doped ZrO_2_ crystals are excited with a 980 nm DPSS laser, mostly green UC is generated, which decrease along with the lanthanide ion loading. This observation suggests that, at least at low dopant concentrations, the ESA mechanism contributes to the green UC of the co-doped systems, and it is maybe the reason why the green UC is more intense in ZEY2, the lowest loading sample.

**Figure 5:**
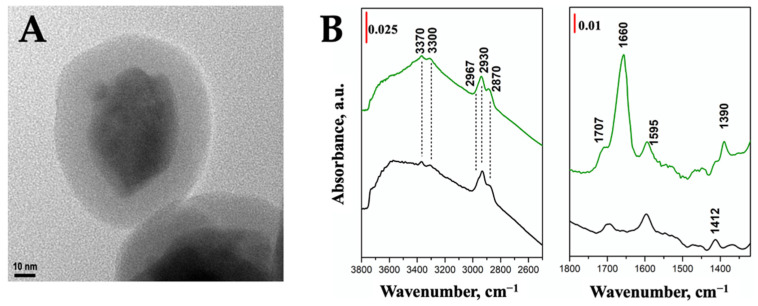
(**A**). TEM image of ZEY2@SiO_2_; (**B**). FTIR spectra of ZEY2@SiO_2_ (black curve) and ZEY2-Ver (green curves).

In order to provide the UC nanoparticles with functional groups for the covalent anchoring of the photosensitizer, amino-functionalized silica shell was deposited on ZEY2 through a facile sol-gel procedure by using TEOS (tetraethylorthosilicate) as the main silica precursor and APTES (3-aminopropyltriethoxysilane) as the functionalizing agent. Verteporfin (Ver) was covalently bound to the amino-modified silica shell through amide bond formation. A silica shell of ca. 20 nm was successfully deposited as confirmed by TEM imaging (Figure 5A). The surface features of the silica coated and Ver-labelled samples (ZEY2@SiO_2_ and ZEY2-Ver, respectively) were investigated by FTIR spectroscopy (Figure 5B).

**Figure 6:**
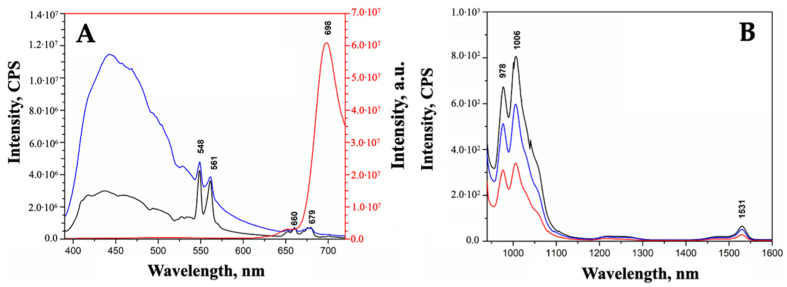
Fluorescence spectra of ZEY2 (curve a, black), ZEY2@SiO_2_ (curve b, blue), and ZEY2-Ver (curve c, red) in the Visible region (**A**) upon excitation at 377 nm and in the NIR region (**B**) upon excitation at 910 nm.

The high frequency region of the FTIR spectrum of ZEY2@SiO_2_ (Figure 5B, black curves) is characterized by signals at 2967, 2930, and 2870 cm^−1^, ascribed to stretching modes of the methylene and methyl groups of the aminopropyl chain and of not completely hydrolyzed alkoxy groups, respectively. Symmetric and asymmetric stretching modes of APTES amino groups are responsible for the signals at 3370 and 3300 cm^−1^, respectively, whilst the corresponding bending mode is visible in the low frequency region of the spectra (1595 cm^−1^), where the signal of the C-N stretching mode is also visible at 1412 cm^−1^. The successful covalent immobilization of Ver was confirmed by the presence, in the ZEY2-Ver FTIR spectrum (Figure 5B, green curves), of the signals at 1390 cm^−1^, due to C-N stretching mode of amide II and of the bands at 1660 and 1595 cm^−1^ ascribed to C-O stretching and N-H bending modes of amide bond, respectively [41,47]. It is worth noting that residual free aminogroups are still present after Ver immobilization, as testified by the presence of signals of NH_2_ stretching modes at 3370 and 3300 cm^−1^. DR UV-Vis-NIR spectrum of ZEY2-Ver (Figure S4 in the Supplementary Materials) is dominated, in the UV and Vis regions, by the intense absorption bands of the PS. Ver spectrum, in fact, is characterized by Soret and Q bands with extinction coefficients much higher than those of lanthanides, therefore no signals from the ZEY2 matrix are visible in the UV-Vis region. On the contrary, as no contributions from Ver are expected in the NIR region, the signals due to Yb^3+^ at 910 and 970 nm are observed. As already observed [48,49], as a consequence of the interaction with solid supports, the absorption spectra of Ver undergo some modifications: beside a general broadening of all the components, a significant increase in the relative intensity of the Q bands (e.g., the component at 690 nm, of interest for the photodynamic activity) with respect to the Soret band is observed. Vis and NIR emission spectra of ZEY2@SiO_2_ and ZEY2-Ver are reported in Figure 6; after the coating with a silica shell, the Er^3+^ green and red emissions are still visible upon excitation at 377 nm. When Ver is present, its emission band, centered at 698 nm, dominates the whole spectrum; this emission profile is compatible with the presence of Ver in monomeric form, as aggregates usually display red-shifted emission bands (>750 nm). This evidence suggests a uniform distribution of the Ver on the silica surface, with a low degree of aggregation. This aspect is very important, Ver being a hydrophobic PS which easily forms aggregates, which are detrimental for the PDT activity as aggregation phenomena severely perturb the ISC (Inter System Crossing) and the triplet excited state yields [50,51].

NIR emission spectra (Figure 6B), obtained upon excitation of the samples at 910 nm, are not perturbed either by the deposition of the silica shell or by the anchoring of the PS, as expected, given that Ver has no emission in the NIR region of the spectrum.

**Figure 7:**
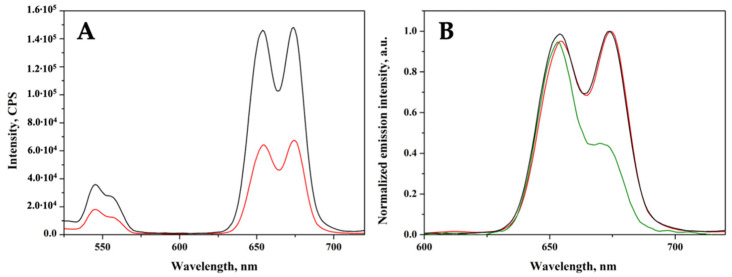
(**A**). UC emission spectra of ZEY2 (curve a, red) and ZEY2@SiO_2_ (curve b, black); (**B**). Normalized UC emission spectra of ZEY2 (red curve), ZEY2@SiO_2_ (black curve), and ZEY2-Ver (green curve).

In order to verify the effect of the silica coating and of the PS anchoring on the UC properties of the co-doped system, UC emission spectra were acquired in the same conditions applied for the pristine co-doped ZrO_2_ nanoparticles, showing that mostly red UC is generated (Figure 7). The deposition of a silica shell has a beneficial effect on the UC properties of the ZEY2 sample (Figure 7A); the intensity of the UC spectrum of the ZEY2@SiO_2_ sample, in fact, is much higher than those of the pristine material. This is in line with reports in the literature about the reduction in surface quenching of UC nanoparticles by the deposition of a passivation layer. The deposition of an inert layer (e.g., SiO_2_), in fact, helps in reducing/blocking energy migration, thus shielding surface quenching sites [52,53,54,55]. In Figure 7B, the UC spectra of ZEY2, ZEY2@SiO_2_, and ZEY2-Ver are reported after normalization in order to highlight the differences in the spectral profile of the red UC bands arising after the immobilization of Ver. Whilst in both the pristine and silica coated spectrum the band at 678 nm and the band at 654 nm has similar intensity (I_654_/I_678_ = 0.98), an inverse trend is exhibited by the ZEY2-Ver sample, where intensity at 654 nm is higher than at 678 nm (I_654_/I_678_ = 2.11). Interestingly, the Q band of Ver, which is excited to trigger PDT activity of the porfin, lies within this spectral range (660–700 nm). Therefore, it is possible to hypothesize that part of the radiation emitted by the Er^3+^ ion is absorbed by the PS; due to the presence of the silica insulating layer, we exclude the possibility of a non-radiative energy transfer from the Er^3+^ to the Ver.

**Figure 8:**
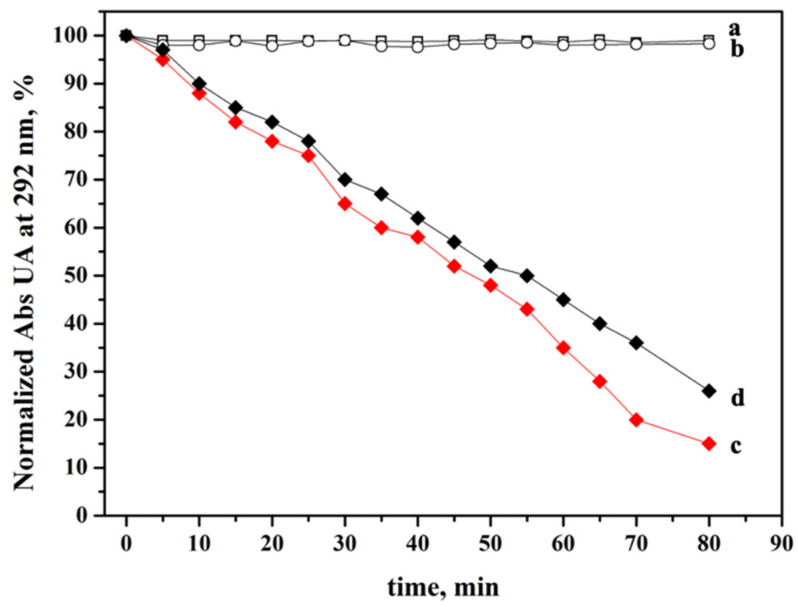
Decrease in the absorption band at 292 nm of uric acid (UA) as a function of the irradiation time, in the presence pristine ZEY2 (curve a, black empty squares), ZEY2@SiO2 (curve b, black empty circles), and ZEY2-Ver under conventional irradiation (curve c, red full squares), or upconversion irradiation (curve d, black full squares).

The photodynamic activity of Ver immobilized on ZEY2 nanoparticles was evaluated by an indirect chemical method using uric acid (UA) as scavenger molecules which undergo oxidation during illumination of the PS, if reactive oxygen species are released [56,57]. Aqueous suspensions of pristine and modified ZEY2 were irradiated at 690 nm and the band decay due to UA oxidation was monitored spectrophotometrically for 80 min, as depicted in Figure 8. The irradiation of ZEY2 and ZEY2@SiO_2_ sample does not cause any significant modification of the UA absorption band, as expected (curve a and curve b, respectively). On the contrary, when Ver is present (ZEY2-Ver sample, curve c), singlet oxygen is efficiently generated and a rapid degradation of UA is observed.

As upconversion measurements (Figure 7B) suggested the possibility of direct excitation of the PS by the red emission of Er^3+^, the photodynamic activity under upconversion conditions was tested, showing that the photodynamic activity of ZEY2-Ver can be efficiently triggered by upconversion (Figure 8, curve d).

## 3. Discussion

Previous investigations on ZrO_2_ nanocrystals and other oxidic matrices doped with a single lanthanide dopant [41,58] evidenced that lower dopant concentrations (namely ≤ 2%) ensure optimal fluorescence performances; therefore, in the design of the co-doped systems, the loading of the acceptor ion, Er^3+^, was kept at a fixed value of 0.5% whilst the concentration of the sensitizer ion, Yb^3+^, was increased from 2% to 10%. Three co-doped samples were prepared, namely ZEY2, ZEY5, and ZEY10, whose composition is given in Table 1 and was confirmed by EDX analysis. When no lanthanide dopants are added to the reaction mix, the synthetic method applied [40,41] allows us to obtain ZrO_2_ nanocrystals where the monoclinic phase is the most abundant; irrespectively of the lanthanide ion nature, as long as the dopant concentration increases, the ZrO_2_ crystalline phase changes from monoclinic to tetragonal. Consequently, along with the increase in dopant loading (e.g., passing from ZEY2 to ZEY5 and to ZEY10) ZrO_2_ nanocrystal population became more and more heterogeneous, being mainly constituted of anisotropic monoclinic phase crystallites (ca. 50 × 20 nm) at low dopant concentration and of a mix of monoclinic crystallites and small particles typical of the tetragonal phase at higher loading.

The characterization of the fluorescence properties of the co-doped systems revealed that another detrimental effect of the increase in dopant loading is the significant concentration quenching, affecting both Er^3+^ Vis emission and Yb^3+^ NIR emission, as already reported both for materials doped with a single lanthanide dopant [41,59] and for co-doped systems [50,60]. The evaluation of upconversion properties, upon excitation at 980 nm, evidenced that mostly red emission of Er^3+^ is generated by upconversion, whilst the intensity of green emission is almost negligible. This aspect is very interesting because red upconversion luminescence is particularly desirable in biological and biomedical applications, but usually, whilst green upconversion is readily obtained in rare-earth doped crystals containing Er^3+^ as the acceptor ion, additional doping or thermal annealing treatments are often needed to increase red upconversion [31,44].

**Figure 9:**
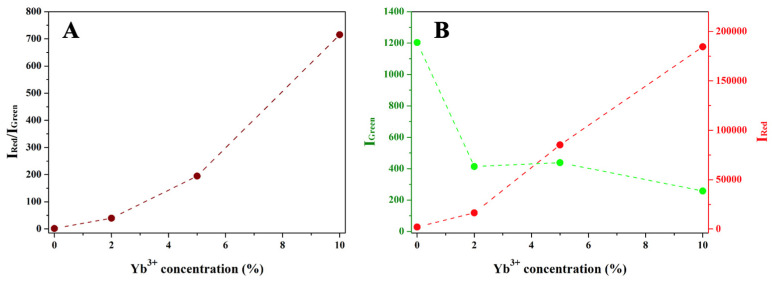
Variation of I_Red_/I_Green_ value (**A**) and of I_Red_ and I_Green_ intensities (**B**) with the change in Yb^3+^ loading; (I_Red_ and I_Green_ represent the integrated intensities of red and green UC emission, respectively).

As evidenced in Figure 9A, the I_Red_/I_Green_ ratio, calculated as the ratio between the integrated area of the red and green upconversion emission bands, increases from 40 to a maximum value of 715 when passing form ZEY2 to ZEY10 sample. As a reference, in Figure 9A, the value of 1.76, obtained when no Yb^3+^ is present, is also reported. It is worth noting that the increase in I_Red_/I_Green_ ratio is the result of the combination of an increase in red upconversion intensity and a decrease in green upconversion (Figure 9B). When only Er^3+^ is present as dopant in ZrO_2_ nanocrystals, in fact, green UC accounts for about 30% of the overall UC emission; the relative intensity of green UC significantly decreases along with the increase in the Yb^3+^ content. As discussed in Section 2, this evidence suggests that the ESA mechanism of UC between neighboring Er^3+^ ions can be responsible for the green UC in low lanthanide loading samples.

**Figure 10:**
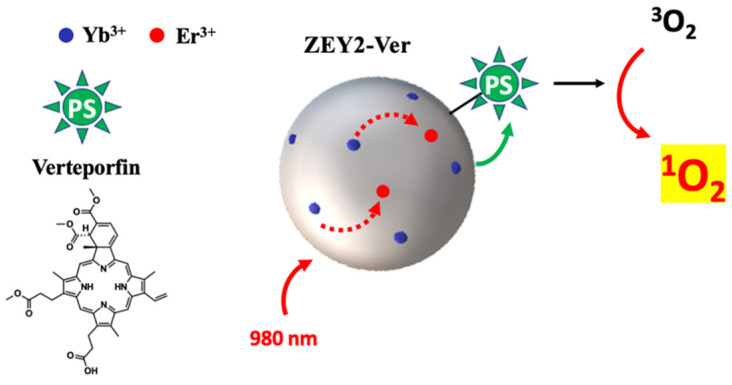
Pictorial representation of the photodynamic activity of Ver triggered by upconversion.

Although red UC is maximized in ZEY10 samples, ZEY2 sample was chosen for the further functionalization because, as discussed above, it is characterized by a high degree of crystals homogeneity, being mainly constituted of anisotropic monoclinic phase crystallites (ca. 50 × 20 nm), it does not suffer from luminescence quenching and its luminescence performances are very good both upon conventional and upconversion excitation. The successful coating of ZEY2 with an amorphous silica shell through a facile Stöber-like procedure [61] was assessed by TEM imaging and FTIR spectroscopy, which confirmed the presence of surface aminopropyl chains as well. The deposition of a silica shell, besides providing anchoring groups for the further functionalization of ZrO_2_ crystals, has a beneficial effect on the UC intensity of ZEY2 sample, which increases after the deposition of silica, as a consequence of the reduction in surface quenching, as already reported in the literature. Aminogroups were used as anchoring groups for the covalent immobilization of Verteporfin through amide bond formation. FTIR and UV-Vis absorption and emission electronic spectroscopies were employed, respectively, to confirm the effective covalent anchoring of the PS and its homogeneous distribution, free from the formation of aggregates, which would be detrimental for the PDT activity. A particular UC behavior was exhibited by ZEY2-Ver: the red upconversion emission in presence of the photosensitizer appears to be perturbed, with a decreased intensity of the portion of the spectrum corresponding to the absorption band of Ver (660–700 nm). This behavior suggests the possibility to push the upconversion form Yb^3+^ to Er^3+^, and further to Ver, thanks to a favorable energy levels match. In fact, beside the conventional excitation with red light, photodynamic activity of Ver was successfully triggered by upconversion, as depicted in Figure 10. Among the visible wavelengths, red light ensures deeper tissue penetration and limited interference from endogenous chromophores, making formulations based on red light absorbing photosensitizers particularly attractive for in vivo PDT applications. Verteporfin and talaporfin, second generation photosensitizers belonging to the family of benzoporfins, are clinically approved and used for the treatment of age-related macular degeneration and lung cancer, respectively. The possibility to trigger the PDT activity by upconversion is particularly interesting because it allows us to further shift towards to the NIR a reactivity that, for organic PS, is limited to the Visible region, thus potentially increasing the possible applications of red light absorbing PS. It is worth noting that ZEY2-Ver displayed PDT activity, which is superior to that exhibited in the same conditions (evaluation of PDT by chemical method, upon excitation with red light) by theranostic systems where Ver was anchored on mesoporous silica nanoparticles [51,62]. These systems were efficient in inducing cell death by PDT in highly metastatic melanoma cancer cells after a few minutes of irradiation (70% of reduction in cell proliferation upon 180 s of irradiation) [63]. Furthermore, in vivo studies carried out on mice with induced melanoma (by the subcutaneous injection of B16-F10 cells) evidenced that a significative reduction in tumor mass, micrometastasis, and lymphoangiogenesis was obtained after 10 min treatment, repeated four times (4 days interval) [64,65]. Such results, obtained with systems characterized by similar performances in the chemical methods, are encouraging for the future application of ZEY2-Ver in both in vitro and in vivo studies.

## 4. Conclusions

In conclusion, UC nanoparticles, characterized by red upconversion emission and particularly interesting for biological applications, have been prepared and subsequently modified by the covalent anchoring of Ver, a photosensitizer which is able to exert its photodynamic activity upon excitation with red light. The architecture of the theranostic nanoparticle ZEY2-Ver, thanks to a favorable energy level match and a uniform distribution of the PS, allowed us to trigger the photodynamic activity of Ver by upconversion, thus paving the way to the use of PDT in deep tissues, thanks to the higher penetrating power of NIR light.

## 5. Materials and Methods

All reagents and solvents were purchased by Merck (Milano, Italy) and used as received.

### 5.1. Synthesis of Er^3+^/Yb^3+^ Doped ZrO_2_ Nanocrystals

Samples of general formula Er_x_Yb_y_Zr_1-(x+y)_O_2_ (where x = 0.005 and y = 0.02; 0.05; and 0.10) were prepared by a hydrothermal process starting from 1M aqueous solution of the precursors (ZrOCl_2_∙8H_2_O. Er(NO_3_)_3_∙5H_2_O, Yb(NO_3_)_3_∙5H_2_O) which were mixed in order to achieve the desired stoichiometric ratio. The pH was then adjusted to 11 by the addition of 4M aqueous NaOH and the mixture was transferred in a 125 mL Teflon-lined stainless-steel acid digestor (Parr Instrument, Moline, IL, USA) and heated to 448 K overnight. The obtained precipitates were washed three times with distilled water, dried in an oven at 333 K, and calcined at 773 K for 2 h.

### 5.2. Coating of Er_0.005_Yb_0.02_ZrO_2_ with Amino-Modified Amorphous Silica

The coating of ZEY2 sample with amino-modified amorphous silica shell was carried out as previously reported [41,66] by the hydrolysis and condensation of tetraethyl orthosilicate (TEOS) and aminopropyltriethoxysilane (APTES). Briefly, 0.5 g of ZEY2 were suspended in 250 mL of a 1:4 water:absolute ethanol mixture; good dispersion was obtained upon sonication in ultrasonic bath. Ammonium hydroxide (28–30%, 2.5 mL) was added to the suspension, followed by the dropwise addition of TEOS (500 μL) as the main silicon source and APTES (50 μL) as the source of amino groups for the covalent conjugation of the photosensitizer. The reaction mixture was stirred at r.t. for 6 h and the ZEY2@SiO_2_ material was then recovered through centrifugation (8000 rpm, 10 min) and dried overnight in air at 353 K.

### 5.3. Immobilization of Verteporfin on Silica Coated ZEY2 Nanoparticles

The covalent conjugation of Verteporfin (Ver) on the surface of silica-coated ZEY2 nanoparticles was carried out through the formation of amide bond upon reaction of carboxylic acid functional group of Ver with aminogroups of APTES exposed on the silica shell [62,67].

Briefly, 0.5 g of ZEY2@SiO_2_ were suspended in 20 mL of dimethylformamide (DMF) along with 2 mg of Ver and 1.1 mg of 1-[bis(dimethylamino)methylene]-1H-1,2,3-triazolo [4,5-b]pyridinium 3-oxid hexafluorophosphate (HATU). The mixture was sonicated in ultrasonic bath for 10 min, then 0.5 mL of N,N-diisopropylethylamine (DIPEA) were added and the mixture was stirred at r.t. for 24 h in the dark. The solid was recovered through centrifugation (8000 rpm, 10 min), washed several times with fresh DMF to remove unreacted Ver molecules, and dried overnight in air at 353 K. The actual Ver loading was calculated from the UV-Vis spectra of the Ver eluate after the washing procedures using the Lambert–Beer law (ε_688 nm_= 35,000 M^−1^ cm^−1^ in DMF).

### 5.4. Characterization Methodologies

X-ray diffraction (XRD) patterns were obtained using an ARL XTRA48 diffractometer operating with Cu Kα radiation (λ = 1.54 Ǻ) generated at 40 kV and 40 mA. Diffractograms were registered in the range 10 < 2Θ < 90 with a step size of 2°min^−1^.

Transmission Electron Microscopy (TEM) images were taken with a JEOL 3010 High Resolution Transmission Electron Microscope operating at 300 kV. Samples were prepared by dispersing the material in isopropanol and depositing few drops of the suspension on carbon-coated copper grids.

Fourier Transform Infrared (FTIR) spectra of self-supported pellets were collected under vacuum (residual pressure <10^−5^ Torr) on a Bruker Equinox 55 spectrometer equipped with deuterated triglycine sulfate (DTGS) detector, working at a resolution of 4 cm^−1^, over 64 scans.

UV-Vis absorption and diffuse reflectance (DR) UV-Vis spectra were recorded on a Perkin Elmer Lambda 900 instrument equipped with an integrating sphere for measurements on solid samples in the diffuse reflectance mode. Reflectance spectra were then elaborated using the Kubelka–Munk function.

Photoemission steady state spectra were collected with a Horiba Scientific Fluorolog spectrofluorimeter equipped with a 450 W Xenon lamp and two detectors: a Hamamatsu R928 photomultiplier (PM) for measurements in the Vis range of the spectrum and an LN cooled InGaAs photodiode for measurements in the NIR region. The spectral response was corrected for the spectral sensitivity of the detector.

Upconversion (UC) spectra were collected on the same Horiba Scientific Fluorolog instrument used for the measurements of steady state emission spectra using a 980 nm diode-pumped-solid-state (DPSS) laser with variable power (0–2 W) and a modulation option as the source and the standard R928 PM as the detector.

The production of Singlet Oxygen by pristine and Ver-modified samples was evaluated by an indirect chemical method using uric acid (UA) as the scavenger molecule. In a typical experiment, UA (1.0 × 10^−4^ M aqueous solution) was added to suspensions of the samples in water and the final suspensions were irradiated with a light source at 690 nm (450W Xenon Lamp). After fixed time intervals, absorption spectra were collected using a Perkin Elmer Lambda 900 instrument. The UA scavenger activity was monitored through the decrease in the UA absorption band at 292 nm over the time and the decrease in UA absorption at 292 nm was plotted against the irradiation time.

## Figures and Tables

**Table 1:** Acronyms, composition, and description of the samples prepared and characterized in the present article.

Acronyms	Sample Composition	Sample Description
ZEY2	Er_0.005_Yb_0.02_Zr_1-(0.025)_O_2_	ZrO_2_ doped with Er^3+^ (0.5%) and Yb^3+^ (2%)
ZEY5	Er_0.005_Yb_0.05_Zr_1-(0.055)_O_2_	ZrO_2_ doped with Er^3+^ (0.5%) and Yb^3+^ (5%)
ZEY10	Er_0.005_Yb_0.1_Zr_1-(0.105)_O_2_	ZrO_2_ doped with Er^3+^ (0.5%) and Yb^3+^ (10%)
ZEY2@SiO_2_	Er_0.005_Yb_0.02_Zr_1-(0.025)_O_2_	Silica-coated ZEY2
ZEY2-Ver	Er_0.005_Yb_0.02_Zr_1-(0.025)_O_2_	Ver-modified ZEY2@SiO_2_

## Data Availability

The data presented in this study are available in this paper and Supplementary Materials.

## Supplementary Materials

The following supporting information can be downloaded at: https://www.mdpi.com/article/10.3390/ijms23136951/s1.
