# Supplementary material for: Red Upconverter Nanocrystals Functionalized with Verteporfin for Photodynamic Therapy Triggered by Upconversion
Source: Int J Mol Sci. 2022 Jun 22;23(13):6951. doi: 10.3390/ijms23136951 (PMC9266923; doi:10.3390/ijms23136951)
Supplement: Supplementary file 1 [file ijms-23-06951-s001.zip › ijms-1775043-supplementary.pdf]

Supporting information

Red Upconverter Nanocrystals Functionalized with Verteporfin for Photodynamic Therapy Triggered by Upconversion

Ivana Miletto [1,*], Chiara Gionco [2,†], Maria Cristina Paganini [2], Erik Cerrato [2,†], Leonardo Marchese [3] and Enrica Gianotti [4,*]

1 Dipartimento di Scienze del Farmaco, Università del Piemonte Orientale, Largo Donegani 2/3, 28100 Novara, Italy
2 Dipartimento di Chimica, Università degli Studi di Torino, Via Pietro Giuria 9, 10125 Torino, Italy; c.gionco@inrim.it (C.G.); mariacristina.paganini@unito.it (M.C.P.); e.cerrato@inrim.it (E.C.)
3 Dipartimento di Scienze ed Innovazione Tecnologica, Università del Piemonte Orientale, Viale Teresa Michel 11, 15121 Alessandria, Italy; leonardo.marchese@uniupo.it
4 Dipartimento per la Sostenibilità e la Transizione Ecologica, Università del Piemonte Orientale, Piazza Sant'Eusebio 5, 13100 Vercelli, Italy
* Correspondence: ivana.miletto@uniupo.it (I.M.); enrica.gianotti@uniupo.it (E.G.)
† Present address: INRiM—Istituto Nazionale di Ricerca Metrologica, Strada delle Cacce 91, 10135 Torino, Italy.

Content

Table S1 – Atomic percentage data obtained from EDX analysis of ZEY2, ZEY5 and ZEY10

Figure S1 – Fluorescence emission spectra obtained by using different excitation wavelength

Figure S2 – UC spectra obtained by using two different laser powers

Figure S3 – Predominant Green UC in UC spectra of Er^{3+}-doped ZrO_2

Figure S4 – DR UV-Vis spectrum of ZEY2-Ver

Table S1 – Atomic percentage data obtained from EDX analysis of ZEY2, ZEY5 and ZEY10

Sample	Element line	Weight %	Atomic %	Atomic % on Zr+Er+Yb
**ZEY2**	O	*25*	*0.66*	
	Zr	*71.3*	*0.33*	*97.35*
	Yb	*3*	*0.0073*	*2.15*
	Er	*0.7*	*0.0017*	*0.5*
	total	*100*	*1*	*100*
**ZEY5**	O	*25.4*	*0.67*	
	Zr	*66.1*	*0.31*	*93.63*
	Yb	*7.8*	*0.019*	*5.82*
	Er	*0.7*	*0.0017*	*0.54*
	total	*100*	*1*	*100*
**ZEY10**	O	*20.6*	*0.624*	
	Zr	*63.8*	*0.3303*	*88.58*
	Yb	*15.0*	*0.044*	*10.97*
	Er	*0.6*	*0.0017*	*0.45*
	total	*100*	*1*	*100*

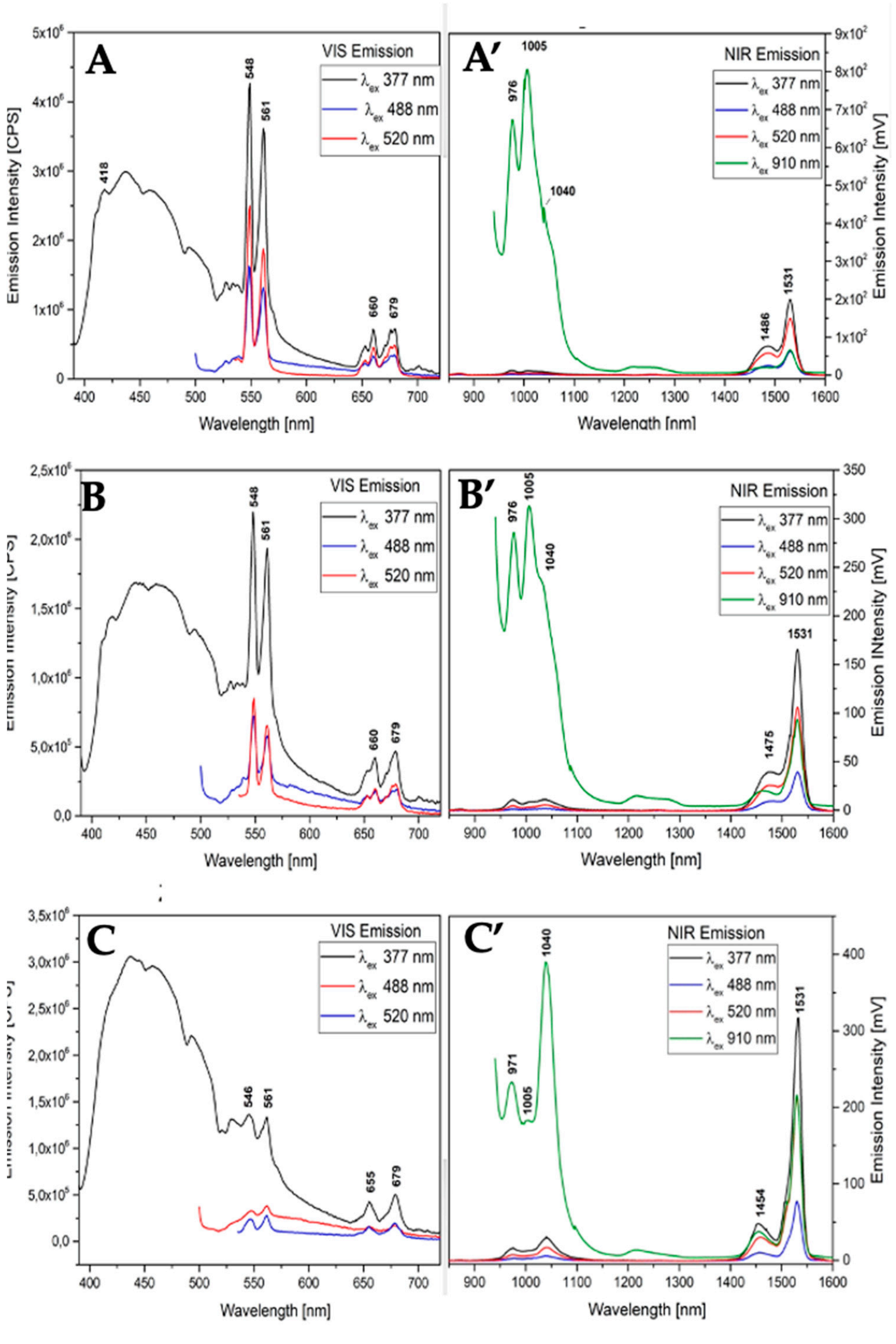

Figure S1 – Fluorescence emission spectra obtained by using different excitation wavelength: ZEY2 (**A**, **A′**); ZEY5 (**B**, **B′**), ZEY10 (**C**, **C′**).

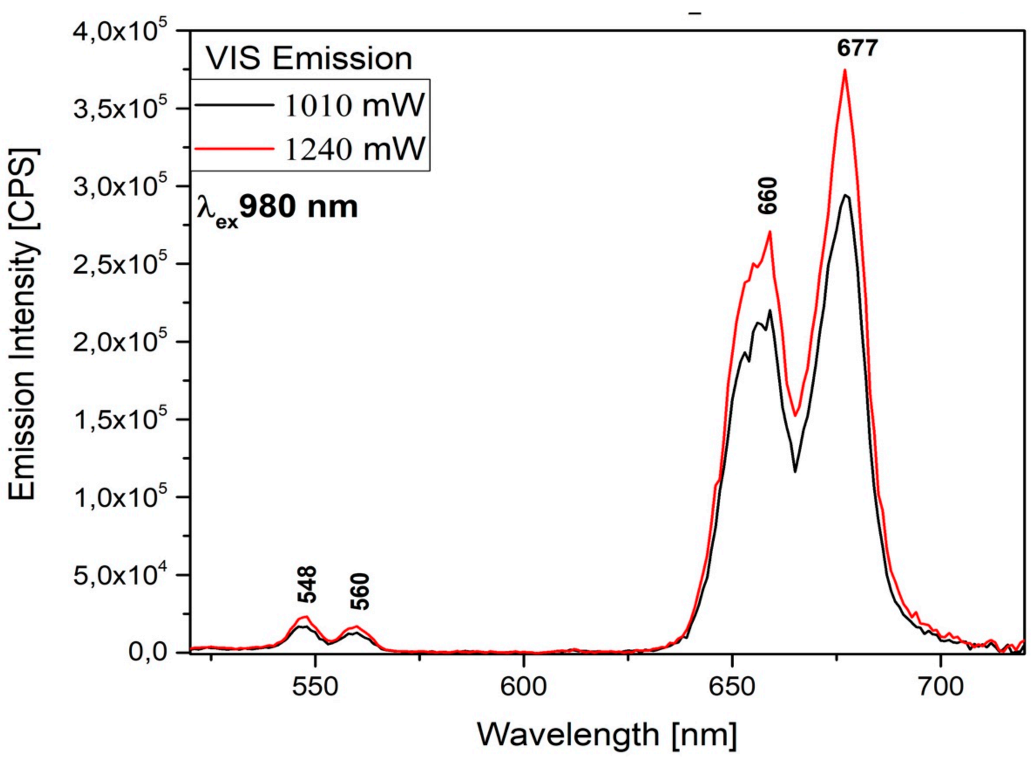

Figure S2 – UC spectra of ZEY2 obtained by using two different laser powers

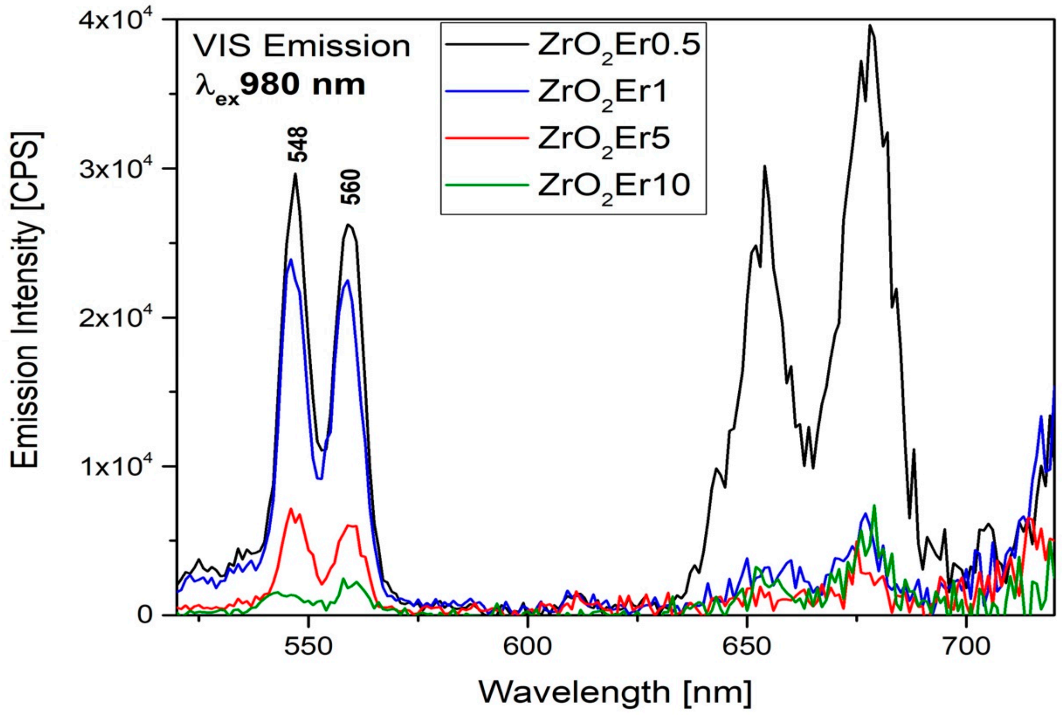

Figure S3 – Predominant Green UC in UC spectra of Er^{3+}-doped ZrO_2

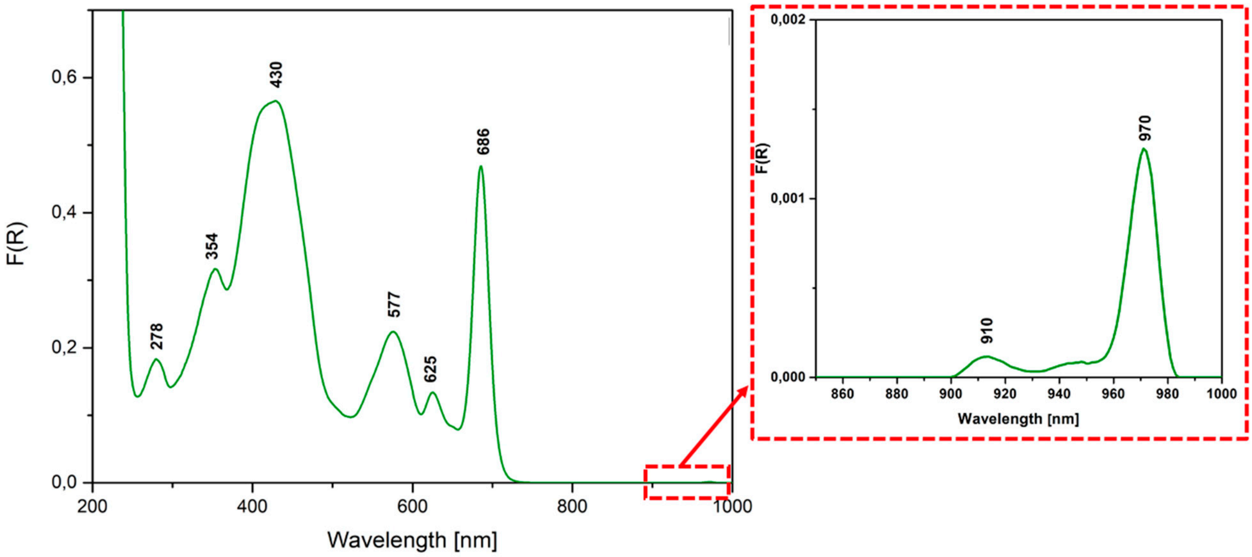

Figure S4 – DR UV-Vis spectrum of ZEY2-Ver
